# A morphologically generalized Eurasian *Pleuroxus aduncus* species group (Cladocera: Chydoridae) is paraphyletic and contains denticulated *P. truncatus*, with an analog in the American *Pleuroxus*

**DOI:** 10.7717/peerj.21616

**Published:** 2026-08-03

**Authors:** Petr G. Garibian, Dmitry P. Karabanov, Anna N. Neretina, Alexey A. Kotov

**Affiliations:** 1 Laboratory for Ecology of Aquatic Communities and Invasions, A.N. Severtsov Institute of Ecology and Evolution of Russian Academy of Sciences, Moscow, Russia; 2 Laboratory of Fish Ecology, Papanin Institute for Biology of Inland Waters of Russian Academy of Sciences, Moscow, Russia

**Keywords:** Molecular phylogeny, Branchiopoda, Taxonomy

## Abstract

Most publications on the water fleas (Crustacea: Cladocera) have focused, and continue to focus, on the genus *Daphnia* O.F. Müller, 1785 which represents a model for several branches of recent biological sciences. In contrast, the number of publications on other numerous genera remains minimal. We have revised the *Pleuroxus aduncus* species group (Anomopoda: Chydoridae), very common in the littoral zone of different continental water bodies of Northern Eurasia. Our study is based on both morphological analysis and molecular phylogenetic data (sequences of the mitochondrial *COI* and *16S*, and nuclear *18S* and *28S* fragments). *P. aduncus* (Jurine, 1820) was redescribed based on European material; a new taxon, *P. korovchinskyi* sp. nov., was described from Eastern Siberia. Our results support the paraphyly of the *aduncus* group and led to a surprising conclusion: *P. truncatus* (O.F. Müller, 1785), a very characteristic taxon having a continuous row of denticles along its posterior valve margin, appeared within the morphologically generalized *P. aduncus* group. This situation has an analog in North America, where the denticulated taxon *P. procurvus* Birge, 1879 also was differentiated within a group of species lacking such denticles at the posterior valve margin. Our study has demonstrated that we need to continue a program of chydorid revisions, as well as of other “widely distributed” cladocerans.

## Introduction

Attempts to describe and systematize freshwater animals have a long history, at least from Aristotle (see Pellegrin, 1986). However, after two millennia of such studies—in the middle of the 20th century—zoology was regarded as an “archaic” science. Indeed, “*the interests of biologists shifted to ecology, biocenology and fish management*” (Korovchinsky, 1997). As a result, taxonomic investigations in continental water bodies were pushed aside as hydrobiology—a new discipline—developed during the 20th century.

New methods of zoological studies proposed at the end of the 20th century—first of all, molecular phylogenetics—has made zoology great again. Such studies were started with “large-sized” animals well-known to a non-scientific public, but then microscopic animals, like water fleas (Crustacea: Cladocera), also were involved in molecular phylogenetic investigations (Hebert & Taylor, 1997; Taylor, Finston & Hebert, 1998). However, most publications on the cladocerans have concerned (still concern) a single genus *Daphnia* O.F. Müller, 1776 (Taylor, Finston & Hebert, 1998; Adamowicz et al., 2009; Bekker et al., 2018; Zuykova et al., 2019) which represents a model for several branches of recent biological sciences (Lampert, 2011; Smirnov, 2017). The number of publications on other, very numerous, macrotaxa are minimal relative to thousands of papers per year devoted to *Daphnia*.

At the same time, in the second part of the 20th century, another water flea group—family Chydoridae Dybowski et Grochowski, 1894—attracted the attention of hydrobiologists due to two main reasons:

(1) This family, being most diverse among the water flea macrotaxa, became a primary model for the radical change of cladoceran biogeography, and then—biogeography of all microscopic inhabitants of the continental waters. David Frey became a proponent of rejection of the cosmopolitanism concept (by followers of Baas Becking’s, 1934) slogan “*Everything is everywhere, but, the environment selects*”). Just chydorids were used by him (Frey, 1982; Frey, 1987) to demonstrate the cladoceran non-cosmopolitanism *via* morphology-based taxonomic revisions of several genera. Chydorid revisions were continued by his followers (Smirnov, 1994; Van Damme, Sinev & Dumont, 2011; Neretina & Sinev, 2016; Sousa et al., 2018; Sinev, Karabanov & Kotov, 2020), these results are reflected in the widely used key books on this family (Smirnov, 1996; Korovchinsky et al., 2021a; Korovchinsky et al., 2021b).

(2) Just family Chydoridae was found to be a valuable model for palaeolimnological studies. After the pioneer paper of Frey (1959), it became obvious that the chydorid remains in bottom sediments could be identified to species level. As a result, cladocerans are regarded now as one of the few model subjects for palaeoecological reconstructions as information on ecological preferences of recent taxa allows us to clarify ecological factors in the past water bodies (Frey, 1962; Frey, 1988a). We need an adequate knowledge of their morphology as their remains are now identified based on morphological characters, although some tests on sedDNA-based cladoceran identification already have been made (Barouillet et al., 2023; Pereboev, Karabanov & Kotov, 2025).

Chydorids are numerous, and their morphology-based revisions in the 21st century mainly involve the subfamily Aloninae Dybowski et Grochowski, 1894 (Van Damme, Kotov & Dumont, 2005; Van Damme, Sinev & Dumont, 2011; Neretina & Sinev, 2016; Sinev, 2020; Sinev, Sousa & Elmoor-Loureiro, 2023), while the number of papers on Chydorinae Dybowski et Grochowski, 1894 (Sinev, Garibian & Gu, 2016; Sousa et al., 2018) is significantly lower. Moreover, only a few publications on chydorids (very rich in species and genera!) incorporate genetic data (Sacherova & Hebert, 2003; Belyaeva & Taylor, 2009; Kotov et al., 2025).

Genus *Pleuroxus* Baird, 1843 is extremely common in fresh water bodies of all continents (except Antarctica, although it is common in Subantarctic islands). Its revision was intensified at the turn of the millennium due to efforts of Frey (1991), Frey (1993a), Frey (1993b), Smirnov (1996), Smirnov, Kotov & Coronel (2006), Smirnov (2007) and Smirnov (2014). It was shown that the genus diversity was greatly underestimated to that time, and it is still underestimated now. Phylogenetic relationships within the genus are almost unknown. Frey (1993b) proposed an inter-generic differentiation based on morphological data, but then its incorrectness was demonstrated (Smirnov, Kotov & Coronel, 2006). Unfortunately, no accurate phylogenetic reconstructions based on genetic data exist to date; preliminary trees (Sacherova & Hebert, 2003) concerned few taxa and have no resolution to resolve any problems of inter-generic relationships.

During the last decade, our team is carrying out a program of the genus taxonomic revision and phylogeny reconstruction combining the morphological and genetic approach (Kotov et al., 2025). The next step of this program is a revision of *P. aduncus* (Jurine, 1820) species group in Northern Eurasia. This is an extremely common species in the littoral zone of many Eurasian water bodies (Smirnov, 2014). Our work combined morphological and genetic approaches and has resulted in acceptance of the *aduncus-*group paraphyly and surprising conclusion: *P. truncatus* (O.F. Müller, 1785), a very characteristic taxon having a continuous row of denticles along its posterior valve margin, falls within the latter. Such an unexpected finding gave us a chance to discuss morphological evolution within *Pleuroxus* in the context of recent chydorid taxonomy.

## Material and Methods

### Ethics statement

Field collection in public property in Russia does not require permissions. No samples were collected in any protected territories. Field works did not affect endangered or protected species.

### Field collection and morphological analysis

Qualitative samples were collected in the littoral zone of the water bodies located in different regions of Russia by dip nets with a diameter of 0.20−0.40 m and a mesh size of 100–250 µm, and immediately preserved in 96% alcohol in the field. The subsequent procedures and morphological analysis methods are described in detail in our previous studies (Garibian et al., 2018; Kotov et al., 2025).

### Abbreviations

**Abbreviations for collections (all specimens are deposited in Moscow, Russia):** AAK, working collection of the Laboratory for Ecology of Aquatic Communities and Invasions, A.N. Severtsov Institute of Ecology and Evolution of Russian Academy of Sciences, Moscow, Russia. MGU, Collection of the Zoological Museum of M.V. Lomonosov Moscow State University.

**Abbreviations used in illustrations and text:** I–V = thoracic limbs I–V; epp = epipodite; ext = exopodite; mxp = maxillar process of limb I; IDL = inner distal lobe of limb I; IP = inter-pore distance (in the head shield); ODL= outer distal lobe of limb I; pep = preepipodite; PP = post-pore distance (in the head shield).

### DNA extraction, sequencing and analysis of sequences

Genomic DNA was extracted from single adult females using the Wizard Genomic DNA Purification Kit (Promega Corp., Madison, WI, USA) according to the manufacturer protocol. Four markers (standard for such studies and used in our previous studies, see Kotov et al., 2025) were investigated here: the 5′-fragment of the first subunit of mitochondrial protein-coding marker cytochrome oxidase (*COI*) (Hebert, Ratnasingham & De Waard, 2003); the 5′-fragment of the mitochondrial *16S* rRNA gene (16S) (Antil et al., 2023); 5′-fragment of the nuclear small (*18S*) and large (*28S*) ribosomal subunits (Reumont von et al., 2009). Primers for their amplification are listed in Table 1.

For amplification of the *COI* fragment for most species, the Fn+Rn primer pair was used (Kotov et al., 2025); in contrast, specimens provisionaly identified as “*Picripleuroxus*” were processed with the Fp+Rp primer pair. For partially degraded samples we used the internal primers for a conservative portion of the *COI* fragment: a combination of F+iR and iF+R primers give two PCR products of about 350 bp length each, with an overlap zone of about 70 bp allowing to form a full contig of *COI* ca. 600 bp.

We used the touchdown PCR protocol (Korbie & Mattick, 2008) to increase the PCR effectiveness. The PCR program included a pre-heating of 3 min at 94 °C; 10 cycles of touchdown (initial denaturation of 30 s at 94 °C, annealing of 30 s at initial temperature +10 °C to optimal one (Table 1), with the hybridization temperature—decreasing by −1 °C per cycle (as a result, after 10 cycles the annealing temperature was equal to optimal one), and elongation of 60 s at +72 °C); 30 cycles of traditional PCR (initial denaturation of 30 s at 94 °C, annealing of 30 s at a specific temperature (Table 1), an extension of 60 s at 72 °C); and a final extension of 7 min at 72 °C. For *COI* fragments, elongation time was halved as compared to traditional protocol.

PCR product was visualized in 1.5% agarose gel and purified according to soft protocol (Karabanov et al., 2022). Each PCR product was sequenced bi-directionally on the “Nanofor-05” analyzer using GenSeq v.1 kit (Volkov et al., 2021) at the Syntol Co, Moscow. Initial analysis of the chromatograms, formation of contigs and their subsequent editing was performed using the Sanger Reads Editor in the Unipro uGENE v.53.1 package (Okonechnikov, Golosova & Fursov, 2012). The authenticity of the sequences was verified by MegaBLAST comparisons (Chen et al., 2015) with published cladoceran sequences in the NCBI GenBank (Sayers et al., 2021) (see detailed information in Table S1). Nucleotide diversity analysis and neutrality tests, namely Fs test (Fu, 1997) and R2-statistics (Ramos-Onsins & Rozas, 2002), were carried out using dnaSP v.6.12 (Rozas et al., 2017).

Alignment of each locus and subsequent analysis of sequences was performed as described in our previous paper (Kotov et al., 2025). Best models of nucleotide substitutions were selected based on the minimum Bayesian information criterion (BIC) (Zhang, Yang & Ding, 2023): *COI* - TVM+F+I+G4, *16S* - TPM2u+F+G4, *18S* - HKY+I and *28S* - TN+F+I.

**Table 1:** Genes, primers and annealing temperatures used in this study.

**Gene**	**Primer**	Sequence 5′–3′	**Temp.** **( °C)**
*COI*	Pl_COI-Fn	CAC TTT AYT TCT TDT TTG GDA TTT G	46
	Pl_COI-Rn	ACG AAA ARA TGT TGA TAW ARA ATA GG	46
	Ple_COI-iF	CGA YTW AAT AAT TTA AGH TTY TGA C	46
	Ple_COI-iR	ATC CHG TWC CWG CYC CTC TYT C	46
	Pl_COI-Fp	TAC TTT ATA CTT CTT ATT TGG	42
	Pl_COI-Rp	AAT AGA TGC TGG TAT AAG ATA	42
*16S*	16Sch-a	GAC TGT GCA AAG GTA GCA TAA TC	52
	16S-br3	CCG GTC TGA ACT CAG ATC ACG T	52
*18S*	18Sa1	CCT AYC TGG TTG ATCCTGCCAGT	52
	18S700R	CGC GGC TGC TGG CAC CAG AC	52
*28S*	28Sd1a	CCC SCG TAA YTT AAG CAT AT	48
	28Sd2b2	CGT ACT ATT GAA CTC TCT CTT	48

Original sequences of our unique haplotypes were deposited to GenBank; accession numbers: PZ037940–PZ038001 (*COI*), PZ049021–PZ049067 (*16S*), PZ049068–PZ049166 (*18S*), PZ049167–PZ049264 (*28S*).

### Phylogenetic reconstructions

Phylogenetic reconstructions based on the maximum likelihood (ML) and Bayesian (BI) methods were made for each gene separately, for the full set of mitochondrial genes, for full set of nuclear genes, and for all unlinked genetic data.

For ML analysis we used IQ-TREE v.3.0.1. Each data set of the sequences was analyzed separately using the best model automatically determined by the ModelFinder with support as 10k replicas in UFBoot2 (Hoang et al., 2018) and SH-aLRT (Guindon et al., 2010) as a test for the tree topology. A clade was considered well-supported if its SH-aLRT >80% and UFboot >95% (Minh et al., 2022).

For BI analysis we used BEAST2 v.2.7.8 (Bouckaert et al., 2019). Parameters of the substitution model, speciation model according to Yule process (Steel & McKenzie, 2001), the Optimised Relaxed Clock model for molecular clocks (Douglas, Zhang & Bouckaert, 2021) for two datasets (two mitochondrial genes—*COI* and *16S*—only and full set of four genes) were identified in BEAUti v.2.7.7 (Drummond et al., 2012), other priors were set up by default (Sarver et al., 2019). We used the *COI* sequence of *Chydorus* KC020624 (Sharma, Elias Gutierrez & Kobayashi, 2014) as an outgroup. In each analysis, we conducted four independent runs of MCMC (100 M generations, with selection of each 10 k generation). Subsequent analysis pipeline is descibed in our previous paper (Kotov et al., 2025).

For preliminary species/phylogenetic lineage delimitation, we subdivided all the sets of sequences into large population groups (clades) based on the “barcode gap” concept and formalized in the “Local Minima” function (locMin) (Rangel-Medrano, Ortega-Lara & Marquez, 2020), which was implemented using the ‘spider’ package (Brown et al., 2012). Most relevant approaches were used for single loci:

(1) identification of vouchers using a search in the Barcode of Life Data System v.5 (Ratnasingham & Hebert, 2007) through the ‘bold_identification’ script (Yang et al., 2020);

(2) LocMin approach itself (Rangel-Medrano, Ortega-Lara & Marquez, 2020);

(3) Assemble Species by Automatic Partitioning (ASAP) method (Puillandre, Brouillet & Achaz, 2021) with determination of gap by locMin (Ota et al., 2020),

(4) An approach based on the population genetic theory, calculations of the K (the average pair distance between putative species-level clades) over Theta (estimation of the genetic diversity) rate performed in the “KoT –K over Theta” (Spori et al., 2021);

(5) bGMYC algorithm (Reid & Carstens, 2012) referred to Yule coalescent models;

(6) same with the Multi-rate Poisson tree processes (mPTP) (Kapli et al., 2017);

(7) multi-species coalescent “Species Tree And Classification Estimation, Yarely” model (STACEY) (Jones, 2017).

A qualitative comparison of alternative partitions was visualized as a heatmap in the ‘ComplexHeatmap’ package (Gu, Eils & Schlesner, 2016) in R version 4.5.1 (R Core Team, 2026). Advantages and disadvantages of each delimitation method and delimitation algorithm are discussed in our previous paper (Karabanov et al., 2023).

The tanglegram was generated using the NN-tanglegram method in SplitsTree v6.7.4 (Huson & Bryant, 2024), which heuristically rearranges taxa to minimize line crossings while preserving the overall tree topology.

## Results

### Morphological account

**Table:** 

**Order Anomopoda Sars, 1865**
**Family Chydoridae Dybowski & Grochowski, 1894 emend. Frey, 1967**
**Subfamily Chydorinae Dybowski & Grochowski, 1894 emend. Frey, 1967**
**Genus *Pleuroxus*** ** Baird, 1843**

**Table:** 

**Figure 1:**
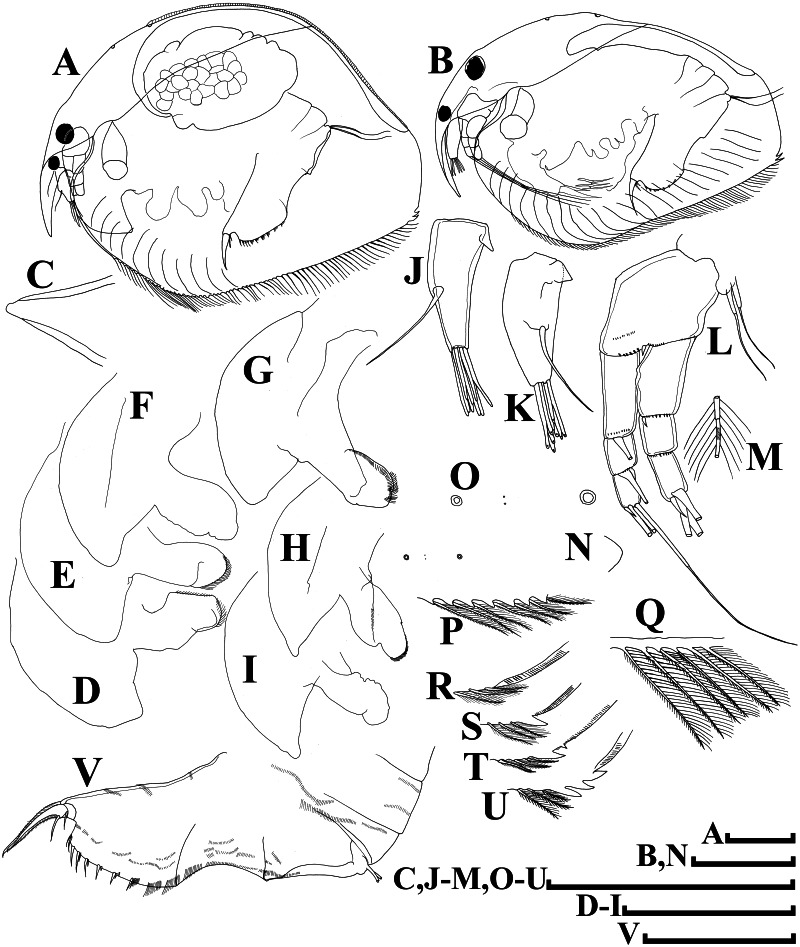
*Pleuroxus aduncus* (Jurine, 1820) parthenogenetic females from European Russia. Specimens from a flooded valley of the Anapka River, Krasnodar Territory (A–H, J–Q, S–W); Bolshoy Irgiz River, Saratov Area (I); Pond in the Ostankino park, Moscow City (R): (A) Adult female. (B) Juvenile female. (C) Rostrum, dorsal view. (D–I) Labrum. (J–K) Antenna I. (L) Antenna II. (M) Swimming seta of antenna II. (N–O) Head pores. (P) Anterior part of valve ventral margin. (Q) Posterior part of valve ventral margin. (R–U) Valve postero-ventral angle. (V) Postabdomen. Scale bars: 0.1 mm.

**Figure 2:**
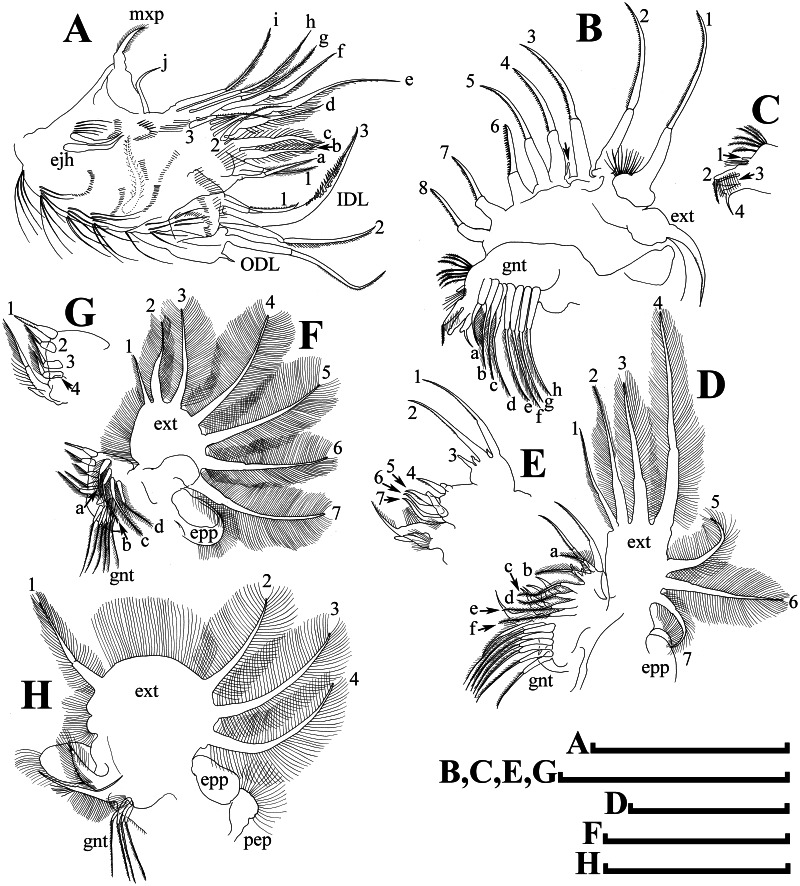
*Pleuroxus aduncus* (Jurine, 1820) parthenogenetic females from European Russia. Specimens from a flooded valley of the Anapka River, Krasnodar Territory: (A) Limb I. (B) Limb II. (C) Gnathobase of limb II. (D) Limb III. (E) Inner portion of limb III. (F) Limb IV. (G) Inner portion of limb IV. (H) Limb V. Scale bars: 0.1 mm.

**Figure 3:**
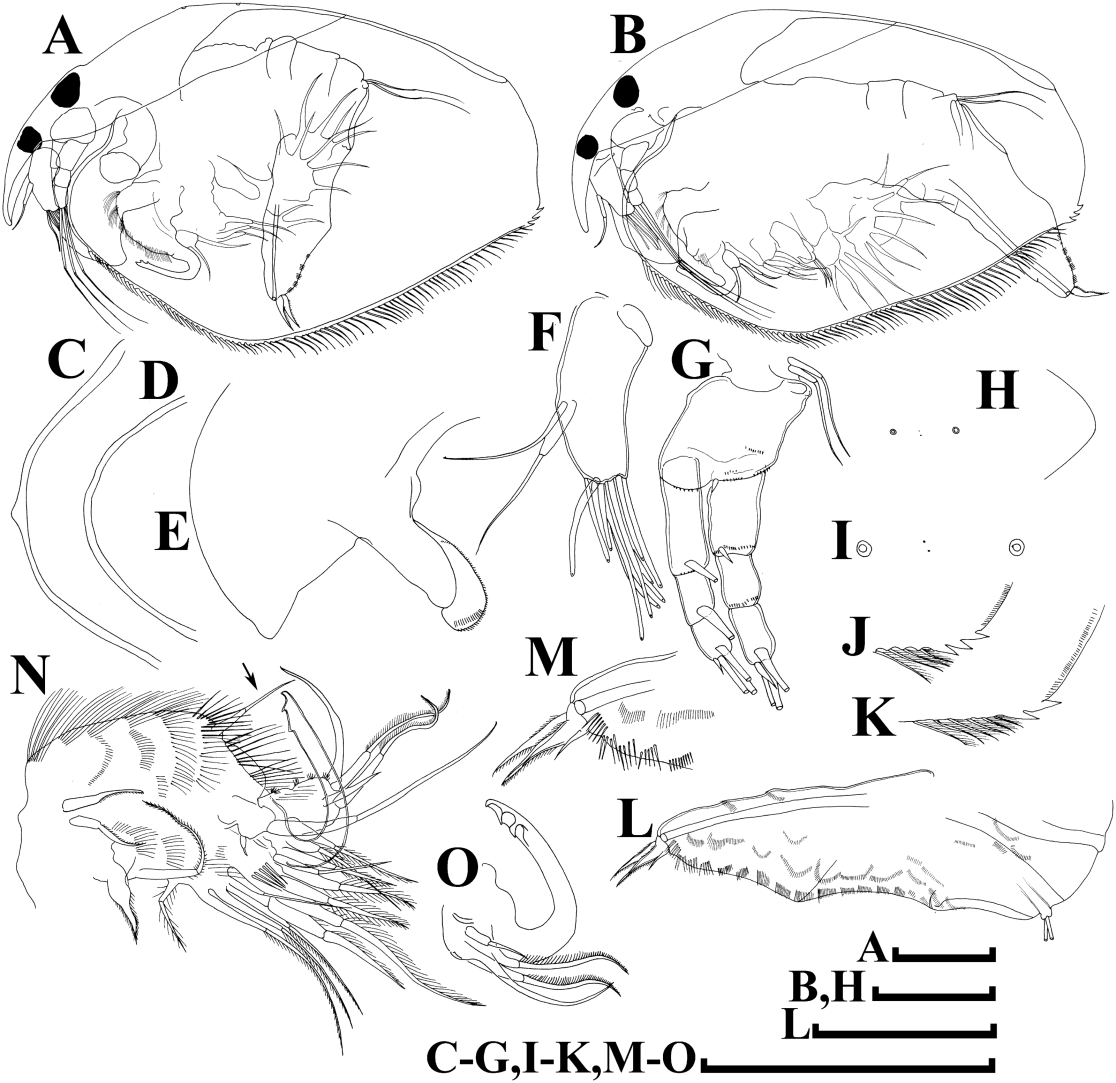
*Pleuroxus aduncus* (Jurine, 1820) adult male from European Russia. Specimens from Udaltsovskiy pond, Moscow City (A, C, E–I, K–O) and a pond near the highway, Rostov Area (B, D, J): (A–B) Adult male. (C–D) Rostrum in dorsal view. (E) Labrum. (F) Antenna I. (G) Antenna II. (H–I) Head pores. (J–K) Valve postero-ventral angle. (L–M) Postabdomen. (N) Limb I. (O), IDL of limb I. Scale bars: 0.1 mm.

***Pleuroxus aduncus*** **(Jurine, 1820)**
(Figs. 1–3)

**Figure 4:**
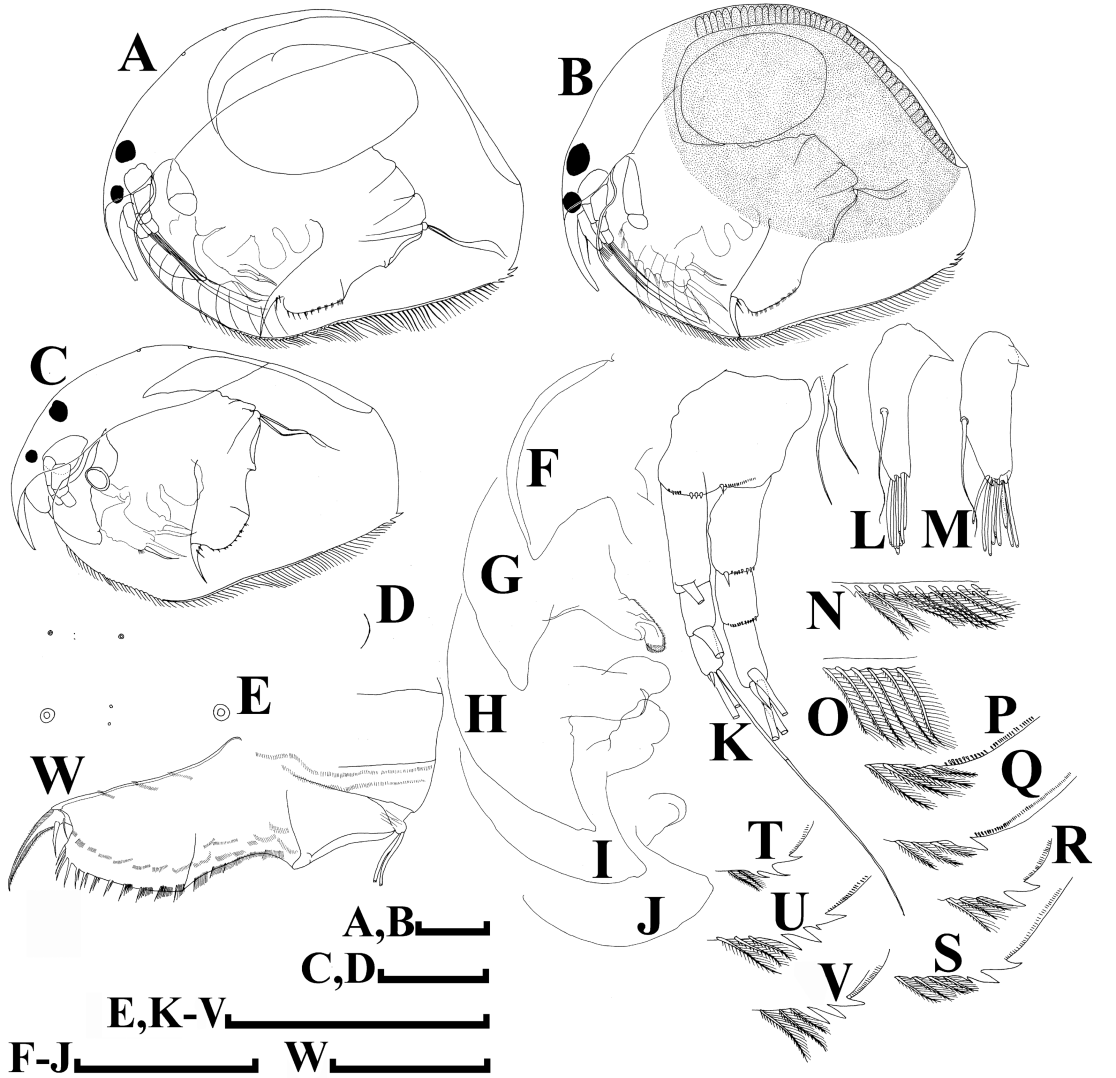
*Pleuroxus korovchinskyi* sp. nov., parthenogenetic female and ephippial female from the Republic of Sakha (Yakutia). Specimens from Lake 4 near the road Amga-Churapcha, Churapcha Ulus (A–B, U); Lake 1 near the road Amga-Churapcha, Churapcha Ulus (C–E, G–I, K–X); Lake near Karbalakh, Tatta Ulus (F, J): (A) Adult parthenogenetic female. (B) Ephippial female. (C) Juvenile female. (D–E) Head pores. (F–J). Labrum. (K) Antenna II. (L–M) Antenna I. (N) Anterior part of valve ventral margin. (O) Posterior part of valve ventral margin. (P–V) Postero-ventral angle. (W) Postabdomen. Scale bars: 0.1 mm.

Jurine, 1820: 152–153, Fig. 7–8 (*Monoculus aduncus*); Lilljeborg, 1901: 541–545, Pl. 75: Fig. 78–81); Behning, 1941: 284–287, Fig. 118; Sramek-Husek, Straskraba & Brtek, 1962: 381–383, Pl. 143; Manujlova, 1964: 238–239, Fig. 122; Scourfield & Harding, 1966: 44, Fig. 100; Smirnov, 1971: 320–325, Fig. 201–206; Flossner, 1972: 359–361, Pl. 169; Negrea, 1983: 237–240, Fig. 95; Margaritora, 1983: 103, Fig. 66A–B; Margaritora, 1985: 208–211, Fig. 83; Frey, 1991: 295–300, Fig. 2–47; Frey, 1993a: 184–185; Smirnov, 1996: 48–50, Fig. 157–193; Alonso, 1996: 266–268, Fig. 119; Flossner, 2000: 255–258, Pl. 95; Hudec, 2010: 415–418, Fig. 102; Kotov et al., 2010: 268, Pl. 151, Fig. 2–4; Rogers et al., 2019: 713, Fig. 16.2.46L–N; Korovchinsky et al., 2021a; Korovchinsky et al., 2021b: 432–433, Fig. 134.

**Type locality.** “Les mares de Châtelaine” (Jurine, 1820), Jura Department in Bourgogne-Franche-Comté, Eastern France.

**Type material.** Presumably lost (Smirnov, 2014).

**Material examined here.** Many parthenogenetic, ephippial females and males from European Russia: Moscow City, Moscow Area, Rostov Area, Volgograd Area, Krasnodar Territory (see Table S1).

**Short diagnosis**. Female body high for genus, transparent, without a dorsal keel and any lateral projections. Rostrum long, acute, directed downwards, or somewhat posteriorly. Head shield elongated, PP approximately 3IP. Apex of distal labral plate with a variable shape: from projected and pointed to shortened and rounded. Postero-ventral angle of valve with a few (0–3) denticles directed somewhat upwards. Postabdomen moderately elongated, narrowing distally, preanal and postanal angles well-expressed, postanal part short, noticeably narrowing distally, with several relatively short postanal teeth, anal portion bearing grouped setules. Postabdominal claw with two basal spines of unequal size. Antenna I elongated, basal peg of moderate size, with blunt tip. Antennal formula: setae 0-0-3/1-1-3; spines 1-0-1/0-0-1. A single exopodite swimming seta shorter and slender than two other setae; each swimming seta with a chitin insertion in its distal segment. Exopodite with a relatively long distal and short proximal spine. Limb I ODL with a long setae setulated distally and a short setae. IDL with three setae of different size and armature: seta 1 shortest, with small setules distally, seta 2 relatively long, setulated distally, seta 3 robust and hook-like, with numerous spines on the concave edge. Filter plate of gnathobase II with 8 setae; gnathobase III with 8 setae; gnathobase IV with 6 setae; gnathobase V with 4 setae.

Male with more elongated body. Rostrum shorter as in female, in dorsal view rostrum ranges from round (in more juvenile forms) to subrectangular (in more adult specimens), it usually has a small, rounded medial projection, but sometimes it is absent. Postabdomen elongated, tapering distally; postanal and preanal angles present, but a bit smooth; postanal and anal portion with groups of setules; distalmost bunches contain longer setules. Gonopores open ventrally at level of postabdominal claw. Antenna I with a basal peg having a blunt tip, and nine aesthetascs of strongly different size: shortest aesthetascs more than two times shorter that longest one.

### Redescription

**Parthenogenetic female**. Body subovoid, high in adults (body height/body length 0.66–0.74, Fig. 1A), but lower in juveniles (Fig. 1B), transparent, lacking a dorsal keel and any lateral projections. It is compressed laterally, maximum height approximately in body middle. Dorsal margin without a depression between head and carapace evenly arched from rostrum tip to postero-dorsal angle. Posterior margin almost straight. Ventral margin rounded in its anterior portion and straight in its posterior portion. Obscure striation as stripes on ventral portion of body.

Head relatively small, with a long, acute rostrum, protruding downward, or somewhat posteriorly. In dorsal view rostrum triangular, with acute tip (Fig. 1C). Head shield elongated, with two major head pores and two minute lateral pores, PP approximately 3IP (Figs. 1N, 1O). Labrum with a fleshy main body and a relatively large, flat distal labral plate, distal angle mostly acute. Apex of distal labral plate with a variable shape: from projected and pointed to shortened and rounded (Figs. 1D–1I).

Valve relatively large (Figs. 1A, 1B), ventral margin convex in its middle; its anterior portion with relatively short setae and posterior portion with long setae (Figs. 1P, 1Q). Postero-dorsal angle rounded, postero-ventral angle expressed and armed with a few (0–3) teeth directed somewhat upwards (Figs. 1R–1U).

Postabdomen moderately elongated, narrowing distally, ventral margin almost straight (Fig. 1W). Preanal margin almost straight, anal margin concave, postanal margin straight, preanal angle well expressed. Postanal part short, noticeably narrowing distally, with several relatively short postanal teeth, anal portion bearing grouped setules. Postabdominal setae short. Postabdominal claw long and slightly curved, armed each dorsally and ventrally with a row of short setules; two basal spines of unequal size: proximal most one relatively short.

Antenna I relatively short, not reaching tip of rostrum, narrowing distally, bearing a well-defined basal peg and nine short aesthetascs distally (Figs. 1J, 1K). Antennal seta as long as antenna body, slender, located at the middle of antennular body. Antenna II (Figs. 1L, 1M) relatively short, coxal part with two sensory setae, basal segment quadrangular, with a small distal spine. Exopod and endopod of antennal branches subequal in size. Antennal formula: setae 0-0-3/1-1-3; spines 1-0-1/0-0-1. A single exopodite swimming seta shorter and slender than two other setae; each swimming seta with a chitin insertion in its distal segment, (Fig. 1M) covered by numerous lateral setules. Exopodite with a relatively long distal and short proximal spine.

Limb I relatively large (Fig. 2A). ODL large, with a long setulated distally setae and a short setae. IDL with three setae of different size and armature: seta 1 shortest, with small setules distally, seta 2 relatively long, setulated distally, seta 3 robust and hook-like, with numerous spines on the concave edge. Endite 3 with three soft posterior setae (a–c) and stiff anterior seta 1 of similar length. Endite 2 three posterior setae (d–f) and selicate anterior seta 2 armed with minute setules. Endite 1 with long, slender posterior setae g–i, seta j of a moderate size, and anterior seta 3 (slightly shorter than seta 2). Fascicles of thin setules on inner face of limb, plus bunches of longer thicker setules at ventral margin of limb. Two slender ejector hooks. Maxillar process (gnathobase) with a single setulated seta.

Limb II (Figs. 2B, 2C) exopodite small, with a single long seta. Inner portion of limb II with eight scraper setae, setae 1–3 especially long, 4–5 setae shorter than previous, setae 6–8 shortest one, subequal in size. Inner limb portion with row of setules near setae 1, and small sensillum near scraper setae 4. Distal armature of gnathobase with four setae: setae 1–2 reduced, setae 3–4 short and setulated. Gnathobase filter plate of eight setae.

Limb III (Figs. 2D, 2E) epipodite ovoid, exopodite large and subrectangular, bearing eight setae of different size, setae 1 covered by short setules distally, setae 4 longest one. Inner-distal limb portion (distal endite) with three anterior setae and two sensilla, seta 1 and 2 relatively long with a short setulation, setae 3 short. Basal endite bearing four short, anterior setae (4–7) subequal in size and two sensilla. Outer portion of limb III with six soft setae (a–f). Distal armature of gnathobase with three setae: first seta relatively long and setulated, two other setae shot and reduced. Gnathobase filter plate with eight setae.

Limb IV (Figs. 2F, 2G) pre-epipodite large and setulated, epipodite large, exopodite ovoid, with seven soft setae, setae 1–3 relatively short (seta 1 the shortest one), setae 4–7 long and subequal in size. Inner-distal limb portion with four short anterior setae, seta 1 without setulation, setae 2–4 setulated. Posterior face of limb IV with four soft setae. Distal armature of gnathobase with two setae, first seta relatively long and setulated, second seta short. Gnathobase filter plate with six setae.

Limb V (Fig. 2H) pre-epipodite small and setulated, epipodite small and rounded; exopodite large and ovoid, with four soft setae (a single distal seta and three lateral setae). Inner-distal portion of limb V with a flat setulated lobe and two setae. Gnathobase filter plate with four setae.

**Ephippial female** not found in our material. According Hudec (2010) maximum length of ephippial female 0.58 mm. Ephippium with a single egg (Korovchinsky et al., 2021b).

**Adult male**. Body (Figs. 3A, 3B) ovoid and elongated (height/length ration 0.61–0.64 in our material), transparent. Maximum height approximately in body middle. Dorsal margin of body slightly convex, posterior margin from almost straight to convex, ventral margin convex in the middle. Postero-dorsal and postero-ventral angles well-visible.

Head relatively small, narrow in lateral view. Distance between eye and ocellus shorter than distance between ocellus and tip of rostrum. Rostrum shorter as in female. In dorsal view rostrum (Figs. 3C, 3D) ranges from round (in more juvenile forms) to subrectangular (in more adult specimens), it usually has a small, rounded medial projection, but sometimes it is absent (Fig. 3C). Head shield (Figs. 3H, 3I) with two major head pores and two lateral head pores, IP distance shorter than PP distance about two times.

Valve (Figs. 3A, 3B) as in juvenile female, elongated and ovoid. Ventral margin of valve covered by row of numerous setae of different size, postero-ventral angle (Figs. 3J, 3K) with a few (2–3) denticles.

Labral keel generally shorter than in female (Fig. 3E)

Postabdomen (Fig. 3L) elongated, tapering distally; its ventral margin almost straight, anal and postanal margins slightly concave. Postanal and preanal angles present, but relatively smooth. Postanal and anal portion with groups of setule bunches; distalmost bunches contain longer setules. Gonopores open ventrally at level of postabdominal claw. Postabdominal claw (Figs. 3L, 3M) short and almost straight, with a single basal spine.

Antenna I (Fig. 3F) cylindrical, thicker than in female, with basal peg of a moderate size and blunt tip. Male seta long (longer than the antennular sensory seta). Male seta and antennular sensory seta located approximately at the middle of antenna I body. Distal part of antenna I with nine terminal aesthetascs of strongly different size: shortest aesthetascs more than two times shorter than longest one. Antenna II (Fig. 3G) as in female.

Limb I (Figs. 3N, 3O) with U-shaped copulatory hook, male seta short and slender (Fig. 3N arrow). IDL with four setae of different size.

**Size.** Parthenogenetic female in examined material 0.43–0.77 mm; juvenile male from male 0.42 mm, adult male 0.46–0.53 mm. Range found here somewhat greater than that reported by Smirnov (1996) and Hudec (2010).

**Distribution.** *P. aduncus* s.lat. was regarded as a cosmopolitan taxon (Smirnov, 1996). Most probably, other, morphologically similar, taxa are present in Australia (Smirnov, 1996) and South Africa (Smirnov, 2014). Unfortunately, we do not have final ideas on the distribution of *P. aduncus* s. str. in Eurasia. Most probably, all European populations (Flossner, 1972; Flossner, 2000; Bledzki & Rybak, 2016; Korovchinsky et al., 2021b) represent just the former. At the same time, populations from East Asia, like Yakutia (see this study), Russian Far East (Kotov et al., 2011; Dadykin et al., 2025), Korea (Jeong, Kotov & Lee, 2014), North China (Ji et al., 2015) and Japan (Tanaka, 1989) most probably belong to *P. korovchinskyi* sp. nov.

**Table:** 

***Pleuroxus korovchinskyi*** ** sp. nov.**

**Species name:** urn:lsid:zoobank.org:act:30B5A385-DBC8-4D36-A262-CFC134C416B1

**Figure 5:**
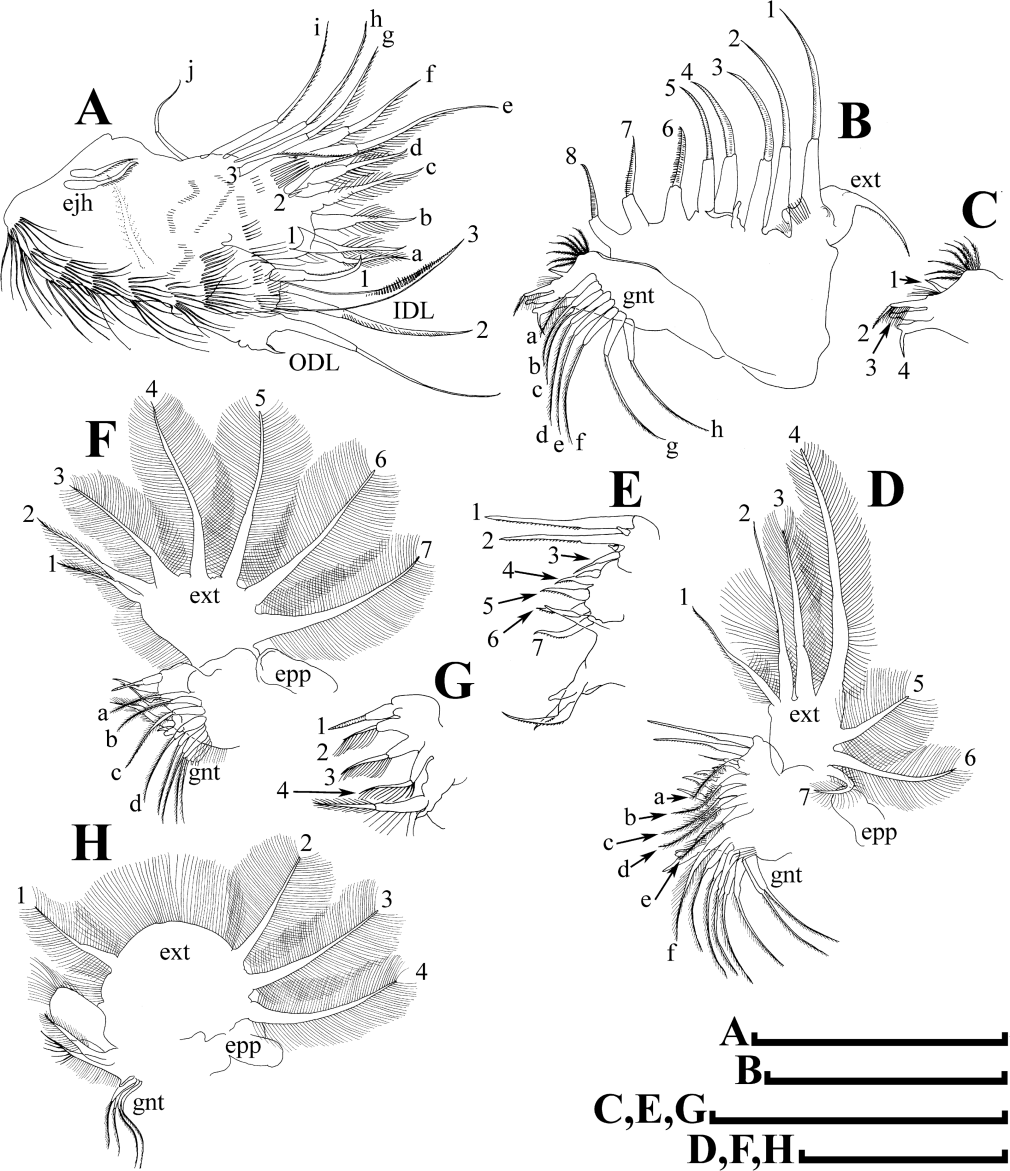
*Pleuroxus korovchinskyi* sp. nov., parthenogenetic female from the Republic of Sakha (Yakutia). Specimens from Lake 4 near the road Amga-Churapcha, Churapcha Ulus: (A) Limb I. (B) Limb II. (C) Gnathobase of limb II. (D) Limb III. (E) Inner portion of limb III. (F) Limb IV. (G) Inner portion of limb IV. (H) Limb V. Scale bars: 0.1 mm.

**Figure 6:**
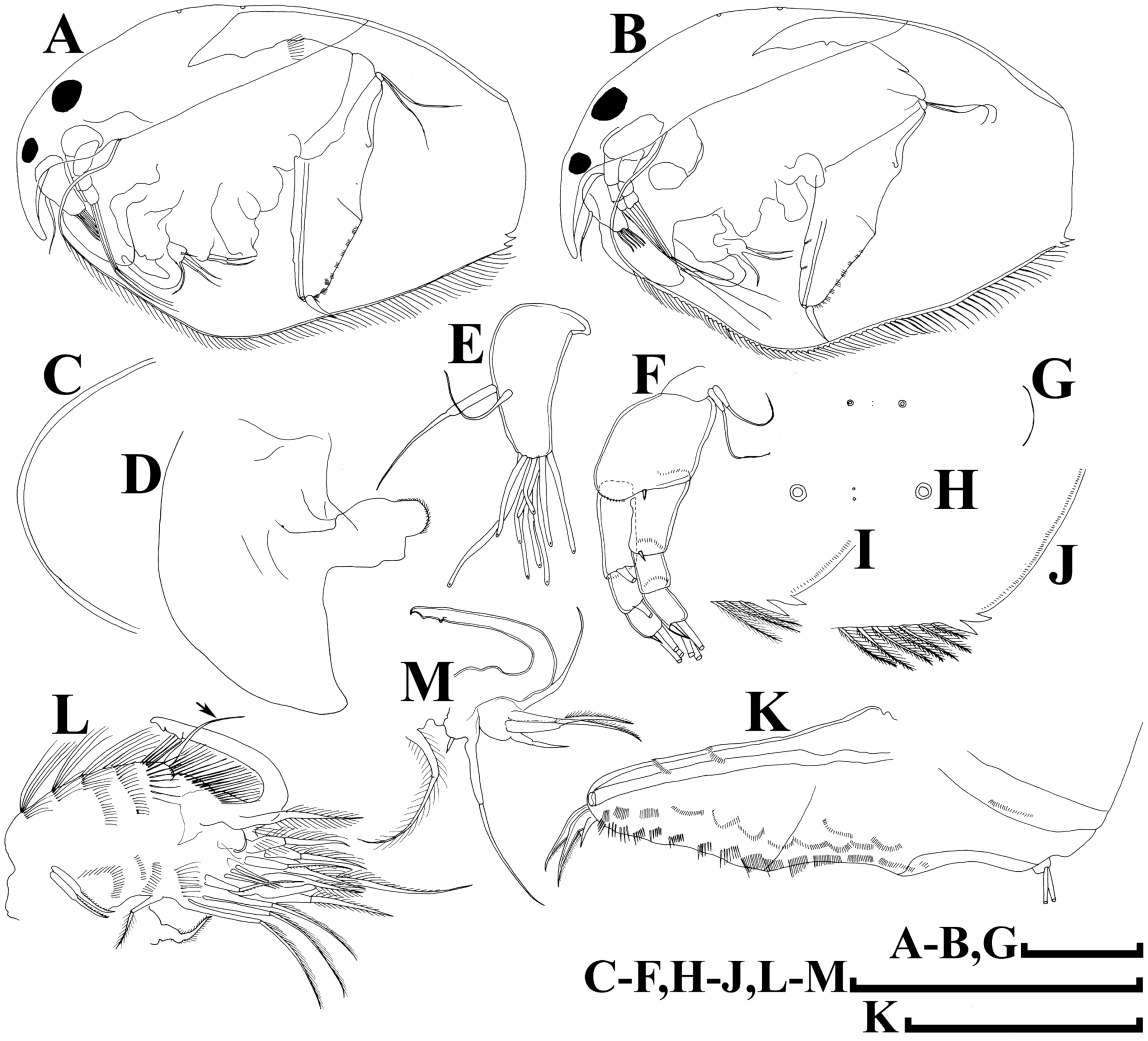
*Pleuroxus korovchinskyi* sp. nov., adult male from the Republic of Sakha (Yakutia). Specimens from Lake 4 near the road Amga-Churapcha, Churapcha Ulus (B–M) and Alasnoe lake, the vicinity of the village of Arylakh, Ust’-Aldan Ulus (A): (A–B) Adult male. (C) Rostrum in dorsal view. (D) Labrum. (E) Antenna I. (F) Antenna II. (G–H) Head pores. (I–J) Valve postero-ventral angle. (K) Postabdomen. (L) Limb I. (M) IDL of limb I. Scale bars: 0.1 mm.

**Publication LSID:** urn:lsid:zoobank.org:pub:52EE9C48-4235-41ED-9A00-5B0623ADAF8 (Figs. 4–6)

**Etymology.** The species is dedicated to our colleague, Dr. Nikolai M. Korovchinsky for his significant contribution to the cladoceran studies in Russia and globally (Korovchinsky, 1997; Korovchinsky, 2018).

**Type locality.** Lake 4 (61.94688°N, 132.6737°E) near the road Amga-Churapcha, Churapcha Ulus, Republic of Sakha (Yakutia), Asian Russia. Type series was collected in 08.2010 by A.A. Kotov.

**Holotype.** An adult male, MGU Ml 292.

**Allotype.** A parthenogenetic female, MGU Ml 293

**Paratypes.** 20 females and a single juvenile male, MGU Ml 294. Many parthenogenetic females and males, AAK M-2003.

**Material excluded from the type series.** Many parthenogenetic females, ephippial females and males from different regions in the Republic of Sakha (Yakutia), see Table S1.

**Short diagnosis**. Female body high for genus (body height/body length 0.63–0.79) in adults (Fig. 4A), more elongated in juveniles (Fig. 4B), transparent, without a dorsal keel and any lateral projections. Rostrum long, acute, directed downwards, or somewhat posteriorly. Head shield elongated, PP∼3IP (Figs. 4D–4E). Apex of distal labral plate with a variable shape: from projected and acute to shortened and rounded (Figs. 4F–4J). Postero-ventral angle of valve with a few (0–3) denticles directed somewhat upwards (Figs. 4N–4W). Postabdomen moderately elongated, narrowing distally, preanal angle well-expressed, postanal angle more smooth relative to *P. aduncus*, postanal part short, noticeably narrowing distally, with several relatively short postanal teeth, anal portion bearing grouped setules (Fig. 4X). Postabdominal claw with two basal spines of unequal size. Antenna I elongated, basal peg well-developed, with a pointed tip (Figs. 4L–4M). Antennal formula: setae 0-0-3/1-1-3; spines 1-0-1/0-0-1. A single exopodite swimming seta shorter and more slender than two other setae; each swimming seta with a chitin insertion in its distal segment (Fig. 4K). Exopodite with a relatively long distal and short proximal spine. Limb I ODL with a long setae setulated distally and a short setae (Fig. 5A). IDL with three setae of different size and armature: seta 1 shortest, with small setules distally, seta 2 relatively long, setulated distally, seta 3 robust and hook-like, with numerous spines on the concave edge. Filter plate of gnathobase II with 8 setae; gnathobase III with 8 setae; gnathobase IV with 6 setae; gnathobase V with 4 setae. All other details (Figs. 5B–5H) as in previous taxon.

Ephippial female shape as in female, only dorsal portion of valves modified into ephippium of a darker color (Fig. 4B).

Male with more elongated body (height/length ration 0.66–0.67 in our material) (Figs. 6A–6B). Rostrum shorter than in female, in dorsal view rostrum round, never has a medial projection (Fig. 6C). Labrum (Fig. 6D) shorter relative to female. Head shield (Figs. 6G–6H), valve (Fig. I–J) as in female. Postabdomen elongated, tapering distally; postanal and preanal angles present, but relatively smooth; postanal and anal portion with groups of setule bunches; distalmost bunches contain longer setules (Fig. 6K). Gonopores open ventrally at level of postabdominal claw. Antenna I with a basal peg having a blunt tip, and nine aesthetascs of different size, but shortest aesthetascs less than two times shorter than longest one (Fig. 6E). Antenna II (Fig. 6F) and thoracic limbs (Figs. 6L–6M) as in previous taxon.

Size: parthenogenetic female 0.36–0.54 mm, ephippial female up 0.55 mm, adult male up to 0.44 mm.

**Differential diagnosis.** The *aduncus*-like populations are different from other Eurasian *Pleuroxus* taxa in: (1) relatively short and high body as compared to “*Picripleuroxus*”, namely, *P. striatus* (Schödler, 1863) s.str, and *P.* cf. *striatus* studied here, *P. laevis* (Sars, 1862), *P. denticulatus* (Birge, 1879), *P. quasidenticulatus* (Smirnov, 1996), but not so rounded as in *P. letourneuxi* (Richard, 1888) and *P. jejuensis* Jeong, Kotov et Lee, 2013; (2) absence of a lateral keel as in *P. smirnovi* Kotov, 2008 or lateral projections on valves as in *P. annandalei* (Daday, 1908) and *P. pamirensis* (Werestschagin, 1923); (3) absence of a tooth row along the posterior margin as in *P. truncatus* (O.F. Müller, 1785); (4) rostrum not directed anteriorly as in *P. uncinatus* Baird, 1850; (5) absence of strong postanal teeth as in *P. trigonellus* (O.F. Müller, 1785) and *P. yakutensis* Garibian et al., 2018.

*Pleuroxus korovchisnskyi* sp. nov. is very close morphologically to *P. aduncus* s. str., but the former could be different from the latter in few characters:

(1) acute tip of the basal peg on female (not male!) antenna I, see a blunt tip of this peg in European populations: Frey, 1991: Fig. 15, Hudec, 2010: Fig. 102B;

(2) rounded rostrum, never having a medial projection in the adult male (see blunt, subrectangular rostrum of European populations: Frey, 1991: Fig. 29–30 Flossner, 1972: Fig. 169C; Margaritora, 1985: Fig. 83B; Hudec, 2010: Fig. 102I;

(3) on male antenna I, shortest aesthetasc less than two times shorter that longest one; see, in contrast, shortest aesthetascs ca. 3 times shorter that longest one in a European population described by Frey, 1991: Fig. 31.

Unfortunately, exact mapping of the distributional ranges of *P. aduncus* and *P. korovchinskyi* sp. nov. is impossible because their morphological diagnosing is difficult based on females, but only they are present in 95% of studied samples.

### Genetic account

**Lineage differentiation.** As we have reported above, whole set of *Pleuroxus* sequences was provisionally subdivided into 18 phylogenetic lineages by the locMin method; the threshold value was calculated as 0.026, which is higher than the species divergence threshold in other animals (Machado et al., 2018), therefore, we have apparently avoided an excessive “splitting”. Values of the neutrality tests (Fs>0 and R2≥0, Table 2) suggest a significant genetic differentiation within *Pleuroxus*, *e.g.*, existence of deeply diverged lines and a complicated population structure. Comparable, not contrasting, results of the neutrality tests for different loci could suggest similar demographic processes for different genes within *Pleuroxus*.

**Table 2:** Genetic diversity of *Pleuroxus* (original sequences only). N, number of sequences; n, total number of sites (excluding sites with gaps/missing data); S, number of segregating (polymorphic) sites; Eta, total number of mutations; Hd, genetype diversity; h, number of genetypes; Pi, nucleotide diversity per site; k, average number of nucleotide differences; Fs, Fu’s neutrality statistic (*Fu*, 1997); R2, Ramos-Onsins and Rozas R2-statistic (*Ramos-Onsins & Rozas*, 2002); D, Tajima’s test (Tajima, 1989) (statistical significance: ns, not significant *P* > 0.10).

**Gene**	**Type**	**N**	**n**	**S**	**Eta**	**h**	**Hd**	**Pi**	**k**	**Fs**	**R2**	**D**
*COI*	mit. coding	62	622	243	355	44	0.98	0.155	96.9	2.51	0.197	1.01 ns
16S	mit. rDNA	47	370	175	265	28	0.95	0.185	66.0	5.99	0.185	0.37 ns
18S	nucl. rDNA	99	590	31	37	9	0.68	0.010	6.0	6.46	0.097	−0.48 ns
28S	nucl. rDNA	98	366	10	13	10	0.76	0.005	2.1	−0.56	0.106	−0.41 ns

**Figure 7:**
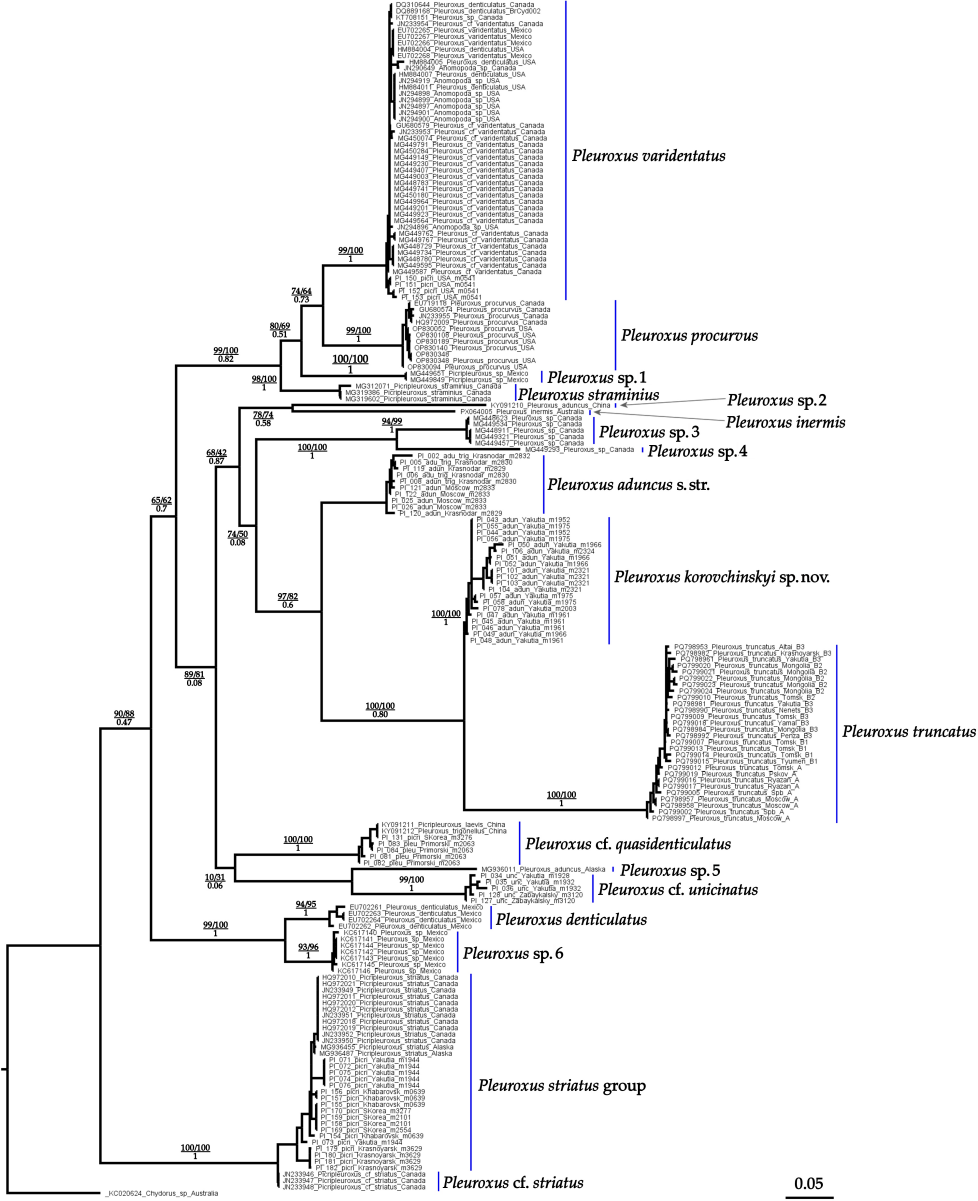
Maximum likelihood tree (IQ-TREE) representing the diversity among phylogroups of *Pleuroxus* based on sequences of mitochondrial gene *COI*. Phylogenetic lineages are separated using locMin method. The support values of individual nodes are based on SH-aLTR/bootstrap UFBoot2 tests (in percent). The bottom number demonstrates the rate of genetic differentiation (*p*-distance).

**COI tree.** Eighteen aforementioned terminal clades are well-distinguishable in the ML tree based on *COI* sequences (Fig. 7).

Five major clades are distinguishable in the *COI* tree: (1) *Pleuroxus* cf. *striatus*; (2) *P. denticulatus* + *P*. sp. 6; (3) *P. varidentatus* + *P. procurvus* + *P*. sp. 1 + *P. straminius* (with a strong support); (4) *P*. cf. *quasidenticulatus* + *P*. sp. 5 + *P*. cf. *uncinatus* (with a very weak support); (5) other species, the “core” group (with a moderate to weak support). We discuss here only the *aduncus*-like clades, they belong just to the “core” (major clade 5). Note that *P. korovchinskyi* sp. nov. is grouped with *P. truncatus* (with 100% support), and this couple makes already a clade with *P. aduncus* s. str. (also with a relatively strong support). Deeper branches in the clade 5 have insufficient support for robust discussion.

We do not discuss here the relationships between major clades of *Pleuroxus* as the former are usually insufficiently supported in the *COI* trees.

Different methods of delimitation represent different number of OTUs. In less conservative variant, 24 clusters are separated because some of 18 aforementioned clades are separated into series on sub-clades (Fig. S1). In most conservative variant, only 13 clusters are separated as: *P. uncinatus, P.* sp. 3, *P.* sp. 4 and *P.* sp. 5 form a *P*. cf. *uncinatus* group; *P. inermis* and P. sp. 2 form a *P. inermis* group. Also, we do not discuss them here; it is important for our purposes that *korovchinskyi* sp. nov., *P. truncatus* and *P. aduncus* s. str. are separated by any delimitation methods.

Tanglegram of the *COI* tree and the tree based on four genes is represented in Fig. S2. There are no contradictions between the trees. Moreover, the clade *P. aduncus* (*P. korovchinskyi* sp. nov. + *P. truncatus*) is well-supported in the four gene BI tree also, and any conflicts between the trees for different genes are absent within this group.

## Discussion

Above we have analyzed both more variable mitochondrial and more conservative nuclear genes, both protein-coding and ribosomal loci, we believe that this allows us to adequately characterize the main *Pleuroxus* phylogenetic lineages. They are present in all trees. Topology of the trees is somewhat different (Fig. S2), but it should be noted that our locus dataset is optimal for the species separation (Karabanov et al., 2023) rather than a full and accurate phylogenetic reconstruction, which is beyond the scope of this study. In any case, our primary aim is to demonstrate the existence of the clade ((*korovchinskyi* + *truncatus*) *aduncus*).

In our study, we follow a more conservative approach in the species delimitation, avoiding excessive “splitting” of phylogenetic lineages into “species”. We do not discuss revealed divergence within the *P. aduncus* and *P. korovchinskyi* sp. nov. clades (*e.g.*, due to a moderate number of studied populations). In our understanding, such intraspecific genetic variation is analogous to that earlier revealed within the *P. truncatus* group (Kotov et al., 2025). We have many different evidences of the new species separation: phylogenetic tree structure, high support values of two different clades, species-delimitation results, genetic distances between the clades, and the limited but available male/female morphological differences, therefore, integrative approach was used in this study.

Above we have found a very obvious difference in mitochondrial and nuclear genes between European and Eastern Siberian populations of “*P.* cf. *aduncus*”. Such east–west genetic differentiation of the cladoceran taxa in Northern Eurasia was demonstrated in many previous publications referred to different cladoceran taxa (Kotov et al., 2016; Zuykova et al., 2019; Sinev, Karabanov & Kotov, 2020) and attributed to the Late Pleistocene history of the continent, *e.g.*, the existing of several glacial refugia in different parts of eastern and western Northern Eurasia. A similar pattern has already been found in two other species groups of the genus *Pleuroxus*, namely the *trigonellus* (Garibian et al., 2018) and the *truncatus* (Kotov et al., 2025) groups. In the latter, it was found that such differentiation is shallow, and establishing of a separate taxon (of species or even subspecies rank) from Siberia seemed unnecessary.

The situation with *aduncus*-like populations differs from previous cases because “the *aduncus* group” is not monophyletic: *P. korovchinskyi* sp. nov. is the closest relative of *P. truncatus* rather than *P. aduncus* s. str. It seems that “*aduncus*” represents an ancestral morphotype lacking any specific morphological structures regarded as diagnostic ones for other *Pleuroxus* groups (see Smirnov, 1971): rostrum curved anteriorly, strong postanal teeth on the postabdomen, a series of denticles at the valve posterior margin, etc. (see differential diagnosis of P. *korovchinskyi* sp. nov.). Possibly, the “*aduncus* group” demonstrates an older phylogeographic pattern, not related to last Pleistocene glaciations considering the great age of the cladoceran macrotaxa (Cornetti et al., 2019; Zharov et al., 2020; Karpowicz et al., 2024), including families and genera of the chydorids (Sacherova & Hebert, 2003).

Our tree clearly indicates that just the *aduncus*-like ancestor (with few postero-ventral denticles on the valve) gave the *truncatus* branch with a continuous row of denticles along the posterior valve margin (see a very high support of the *aduncus*-*korovchinskyi*-*truncatus* clade in the multigene tree, Fig. 7). Denticulated posterior margin is an almost unique feature of *P. truncatus* among *Pleuroxus* taxa, see (Smirnov, 1971). The only North American *P. procurvus* Birge, 1879 has such denticles (in females they are present only in the ventral portion of its posterior margin, but in males—along the full posterior margin), whereas all other species have only few denticles at the postero-ventral angle, or no denticles there (Smirnov, 1971; Smirnov, 1996). Here we clearly demonstrate that the expansion of denticles to the posterior margin occured independently in *P. truncatus* and *P. procurvus* having a very distant position in the phylogenetic trees based on *COI* and four genes. *P. procurvus* is a congener of *P. varidentatus* Frey, 1993 and *P. straminius* Birge, 1879 from New World, also with a high support, see Fig. 7. Note that *P. varidentatus* is an “*aduncus*-like” taxon (Frey, 1993a) , with few denticles at the postero-ventral valve angle only. *P. straminius* has a more elongated body and an elongated, denticle-like postero-ventral angle of the valve (Frey, 1988b). Again, a denticulated taxon was differentiated within a group of species lacking such denticles at the posterior valve margin.

In contrast to the three American taxa (*P. procurvus, P. varidentatus* and *P. straminius*), *P. truncatus* is found to date only in Eurasia. This suggests that denticulated species evolved independently in the Old and New Worlds, therefore, a similar evolutionary pattern was characteristic of non-related species groups from different continents. Such parallel evolution, probably, reflects both adaptations to similar environmental conditions and “design limitations” (Wake, 1991) of such chydorids resulted in similar morphological adaptations within different lineages due to a limited set of enabled modifications of the external morphology.

As in previous studies (Smirnov, Kotov & Coronel, 2006), we do not find a confirmation of the subgeneric differentiation of *Pleuroxus* suggested by Frey (1993b). Indeed, the subgenera according to Frey are mixed in our tree. First of all, the subgenus *P*. (*Peracantha*) Baird, 1843 was suggested by Frey (1993b) for *P. truncatus*, but now we know that this taxon belongs to the *aduncus*-group. Only “*Picripleuroxus* Frey, 1993” which represents a distinct, well-differentiated, group (but does not include *P. denticulatus* and *P. quasidenticulatus*, which were also included in *Picripleuroxus* (Smirnov, 1996). This topic requires further studies in the future.

Previously, several taxa were separated from *P. aduncus* after comprehensive taxonomic revisions (Frey, 1991; Smirnov, Kotov & Coronel, 2006; Smirnov, 2007). Moreover, Smirnov (2014) clearly indicated morphological characters which could be used for a further revision of non-Eurasian populations. At the same time, our study of Eurasian samples led to the conclusion that they are relatively uniform. A partitioning of separate taxa (*e.g.*, of a subspecies rank) within *P. aduncus* s. str. and *P. korovchinskyi* sp. nov. suggested by the formal delimitation methods (Fig. 8) seems to be an excessively “splitting” approach. Our morphological study did not confirm any morphological differentiation within these two taxa.

*P. korovchinskyi* sp. nov. is described here mainly on the basis of molecular genetic data rather than minimal morphological differences revealed above. Note that the minimal set of morphological differences between two taxa described above is already used for differentiation of other chydorid cladocerans (Frey, 1991; Belyaeva & Taylor, 2009). Apparently, such minimal differences between two taxa separated millions of years ago correspond to the concept of a general morphological stasis in the cladocerans (Frey, 1982; Frey, 1987; Zharov et al., 2020). Many reports on the cases where parthenogenetic taxa of congenic species were found to be undistinguishable (or almost undistinguishable) are published to date (Garibian et al., 2018). Here we found a new example of such stasis.

## Conclusions

Our study has demonstrated that we need to continue a program of chydorid revisions, as well as of other “widely distributed” cladocerans. We found that morphologically generalized *Pleuroxus aduncus* species group (Cladocera: Chydoridae), widely distributed and very common in North Eurasia, is paraphyletic and contains denticulated *P. truncatus*, and this situation is similar to that in American *Pleuroxus.* Our genetic data fit well with the Frey’s non-cosmopolitanism concept. A new phylogeny based on genomes could in the future resolve most taxonomic problems and give us a chance to describe evolutionary history and morphological evolution within different chydorid genera.

##  Supplemental Information

10.7717/peerj.21616/supp-1 Supplemental Information 1 BEAST2 tree of *Pleuroxus* based on sequences of four loci (*COI*, *16S*, *18S*, *28S*) with posterior probabilities as the branch support, and conspecificity matrix of its delimitation using seven different me Scale bar in dicates genetic divergence. The heatmap demonstrates a qualitative comparison of alternative partitions from seven different delimitation methods; the color scheme corresponds to the posterior probability within all seven methods. Bars on the right side demonstrate the clusters accepted by at least by four among seven methods (left), and all seven methods (right).

10.7717/peerj.21616/supp-2 Supplemental Information 2 Tanglegram of the multigene BI (left) and ML *COI* (right) phylogenetic trees of *Pleuroxus* Only the topology of the trees is shown, without branch support.

10.7717/peerj.21616/supp-3 Supplemental Information 3 Sequences and samples used in this study. Information on sampled localities is provided

10.7717/peerj.21616/supp-4 Supplemental Information 4 FASTA files and metadata to all gene fragments studies here

## Additional Information and Declarations

### Field Study Permissions

The following information was supplied relating to field study approvals (*i.e.*, approving body and any reference numbers): Field collection in public property in Russia does not require permissions. No samples were collected in any protected territories. Field works did not affect endangered or protected species.

### Data Availability

The following information was supplied regarding data availability: The data is available in the Supplemental Files. The raw sequence data are available at GenBank: PZ037940–PZ038001, PZ049021–PZ049067, PZ049068–PZ049166, PZ049167–PZ049264.

### New Species Registration

The following information was supplied regarding the registration of a newly described species: Publication LSID: urn:lsid:zoobank.org:pub:52EE9C48-4235-41ED-9A00-5B0623ADAF8A Species name: urn:lsid:zoobank.org:act:30B5A385-DBC8-4D36-A262-CFC134C416B1
