# Supplementary material for: A morphologically generalized Eurasian Pleuroxus aduncus species group (Cladocera: Chydoridae) is paraphyletic and contains denticulated P. truncatus, with an analog in the American Pleuroxus
Source: PeerJ. 2026 Aug 3;14:e21616. doi: 10.7717/peerj.21616 (PMC13446229; doi:10.7717/peerj.21616)
Supplement: Supplementary file 1 [file peerj-14-21616-s001.pdf]

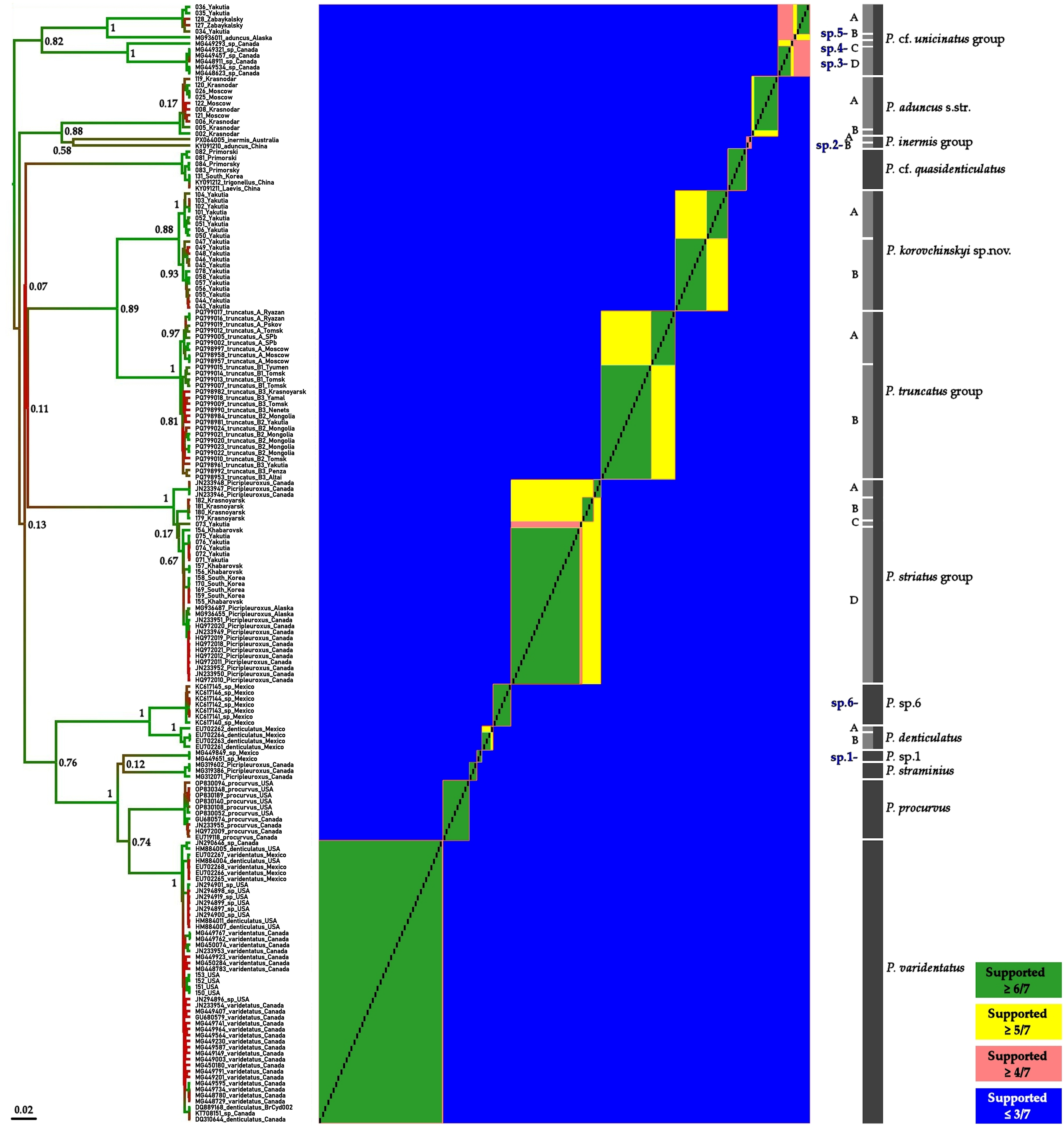

P. cf. unicinatus group
sp.5- B
sp.4- C
sp.3- D
P. aduncus s.str.
P. inermis group
sp.2- B
P. cf. quasidenticulatus
P. korovchinskyi sp.nov.
P. truncatus group
P. striatus group
sp.6-
P. sp.6
P. denticulatus
sp.1-
P. sp.1
P. straminius
P. procurvus
P. varidentatus
Supported ≥ 6/7
Supported ≥ 5/7
Supported ≥ 4/7
Supported ≤ 3/7
0.02
